# Converting three-space matrices to equivalent six-space matrices for Delone scalars in **S^6^**

**DOI:** 10.1107/S2053273319014542

**Published:** 2020-01-01

**Authors:** Lawrence C. Andrews, Herbert J. Bernstein, Nicholas K. Sauter

**Affiliations:** a Ronin Institute, 9515 NE 137th Street, Kirkland, WA 98034-1820, USA; b Ronin Institute, c/o NSLS-II, Brookhaven National Laboratory, Upton, NY 11973, USA; c Lawrence Berkeley National Laboratory, 1 Cyclotron Road, Berkeley, CA 94720, USA

**Keywords:** Delaunay, Delone, centering transformations, centered lattices, reduced cells, lattice centering, Niggli, Selling, matrix transformations

## Abstract

The transformations from the primitive cells of the centered Bravais lattices to the corresponding centered cells have conventionally been listed as three-by-three matrices that transform three-space lattice vectors. Using those three-by-three matrices when working in the six-dimensional space of lattices represented as Selling scalars as used in Delone (Delaunay) reduction, one could transform to the three-space representation, apply the three-by-three matrices and then back-transform to the six-space representation, but it is much simpler to have the equivalent six-by-six matrices and apply them directly. The general form of the transformation from the three-space matrix to the corresponding matrix operating on Selling scalars (expressed in space **S^6^**) is derived, and the particular **S^6^**matrices for the centered Delone types are listed. (Note: in his later publications, Boris Delaunay used the Russian version of his surname, Delone.)

## Abstract 2

Given a matrix for transforming vectors in the three-space of unit-cell edge vectors, the corresponding matrix to transform vectors in the six-space of Delone scalars is derived.

## 1. Introduction   

The transformations from the primitive cells of the centered Bravais lattices to the centered cells and between alternative unit cells have conventionally been listed as matrices that are applied to three-space lattice vectors (Burzlaff & Zimmermann, 1985 ▸; Burzlaff *et al.*, 1992 ▸). However, for both the major cell reductions [Niggli (1928 ▸) and Delone (1933 ▸)], it is convenient to work in a higher-dimension space than **E^3^**, as reported by Andrews & Bernstein (1988 ▸) for **G^6^** reduction and Andrews *et al.* (2019 ▸) for **S^6^**. Therefore, as we did for **G^6^** (Andrews & Bernstein, 1988 ▸), we need to provide the mathematically equivalent six-by-six matrices for centering in **S^6^**. This reduces the need to convert repeatedly from **S^6^** into three-space vectors, transform the three-space vectors, and then transform back into **S^6^**. We derive the general form and list the particular matrices for converting from the 24 canon­ical Delone types to centered lattices in **S^6^**.

## 2. Background and notation   

### The space 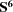   

2.1.

Andrews *et al.* (2019 ▸) introduced the space **S^6^** as an alternative representation of crystallographic lattices. The space is defined in terms of the ‘Selling scalars’ used in Selling reduction (Selling, 1874 ▸) and by Delone (1933 ▸) for the classification of lattices. A point *s* in **S^6^** is defined by
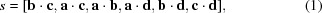
where **d** = −**a** − **b** − **c**.

### The space 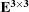   

2.2.

A crystallographic unit cell is commonly represented as three cell edge lengths and three angles, [*a*, *b*, *c*, α, β, γ], but, when presenting common operations on unit cells, it is convenient to express each of the cell edges as a vector in the three-dimensional space of real numbers **E^3^** (also written as **R^3^**) with each cell expressed as a 3 × 3 matrix of real numbers, *i.e.* as an element of **E^3^** × **E^3^** (see Burzlaff *et al.*, 1992 ▸). An issue with this approach is that we should get the same crystallographic unit cell after any proper rotation of **E^3^**, *i.e.* by any unitary matrix of determinant +1. Such proper rotation matrices form the Lie group *SO*(3). Therefore, formally we should treat any matrix representation of a cell *c* ∈ **E^3^** × **E^3^** as equivalent to *rc*, for all *r* ∈ *SO*(3), and work in the space of (**E^3^** × **E^3^**)/*SO*(3). We call this space of equivalence classes **E** ^**3**×**3**^.

Because the matrices that multiply cells represented in **E** ^**3**×**3**^ are indistinguishable from ordinary 3 × 3 matrices, we will designate them as 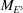, with the understanding that they may be applied in either space **E^3^** or space **E** ^**3**×**3**.^

The convention in **E^3^** is to use the cell edges as the basis vectors of the space. There are infinitely many choices of the orientation to form the basis. Currently, it is the common convention to orient one edge vector along the *x* axis *etc.* in a right-handed setting. The convention in **S^6^** is to use unit vectors [100000], [010000], ….

### 2.3. The method for deriving a transformation in one space from a transformation in another   

Consider two spaces *X* and *Y* with one invertible conversion *M*_*YX*_ mapping
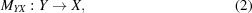
and a not necessarily invertible mapping
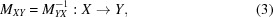
and a transformation of *X* into *X*

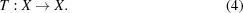
We compose the mappings and the transformation to define a new transformation *U* of *Y* into *Y*

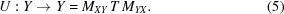
If *Y* is a finite-dimensional linear vector space and *U* is linear, then we can represent *U* as a matrix [https://en.wikipedia.org/wiki/Linear_map] by choosing an appropriate basis. Section A in the supporting information considers the linearity in more detail.

If *X* is in **E** ^**3**×**3**^ and *Y* is in **S^6^**, we can map **E** ^**3**×**3**^ to **S^6^**,
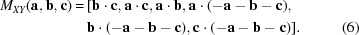
Because *M*_*XY*_ is invariant under rotation, there are infinitely many choices for the inverse. We can choose, for example,
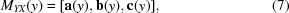
where
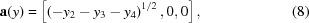


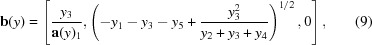


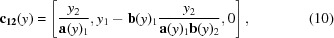


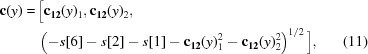
which is applicable for a reasonable set of valid **S^6^** cells. This would then allow a similar demonstration to that given in Section A of the supporting information with the mapping from *Y* to *X* being the simple square root that a linear *T* generates a linear *U* in this more complex but similar case. The details are left as an exercise for the reader.

The point is that, because *U* is linear, the components of its representation as a matrix can be determined by applying it to basis vectors each with only one non-zero component, letting *X* be in **E** ^**3**×**3**^, *Y* be in **S^6^** and *M*_*XY*_ be *E* ^3^ *toS* ^6^ (see Section 3.1).

## Converting an 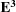 matrix to an 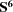 matrix   

3.

If we represent the cell as an **S^6^** vector (Andrews *et al.*, 2019 ▸), we can define an operator *E* ^3^ *toS* ^6^ where *E* ^3^ *toS* ^6^(**a**, **b**, **c**) = [**b** · **c**, **a** · **c**, **a** · **b**, **a** · **d**, **b** · **d**, **c** · **d**], where **d** = −**a** − **b** − **c**.

We form a matrix operating in **E** ^**3**×**3**^, 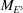 = [[*m*_1,1_, *m*_1,2_, *m*_1,3_], [*m*_2,1_, *m*_2,2_, *m*_2,3_], [*m*_3,1_, *m*_3,2_, *m*_3,3_]]. We need to compute a 6 × 6 matrix, 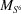, to operate on **S^6^** vectors.

The obvious basis vectors for **S^6^**, [1, 0, 0, 0, 0, 0], [0, 1, 0, 0, 0, 0], [0, 0, 1, 0, 0, 0], [0, 0, 0, 1, 0, 0], [0, 0, 0, 0, 1, 0] and [0, 0, 0, 0, 0, 1], do not correspond to real vectors in **E^3^**, since a dot product of 1 for real non-zero unit basis vectors would imply an angle between them of zero, *i.e.* that they are identical, but if two unit basis **E^3^** vectors, say **a** and **b**, are identical, and one, say **c**, is perpendicular to both **a** and **b**, then the **d** = −**a** − **b** − **c** vector cannot be perpendicular to **a** or **b**, because **a** · **d** = **b** · **d** = −**a** · **a** − **a** · **b** − **a** · **c** = −2**a** · **a**, which cannot be zero. Therefore we use the negatives of those **S^6^** basis vectors.

The **E** ^**3**×**3**^ basis vectors we choose are shown in Table 1 ▸ with the corresponding **S^6^** vectors.

### Relationship to 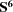   

3.1.

We use the operator *E* ^3^ *toS* ^6^ that converts a vector in **E** ^**3**×**3**^ to one in **S^6^** (see above). In our case, we are starting from reduced unit cells, which means that in **S^6^** all six scalars are zero or negative. We choose the **S^6^** basis vectors to have zero scalars except for a single −1 in each. In **E** ^**3**×**3**^ we choose an orthogonal set (see above), where for each **E** ^**3**×**3**^ vector *E* ^3^ *toS* ^6^ produces only the corresponding **S^6^** basis vector.

**Figure 1:**
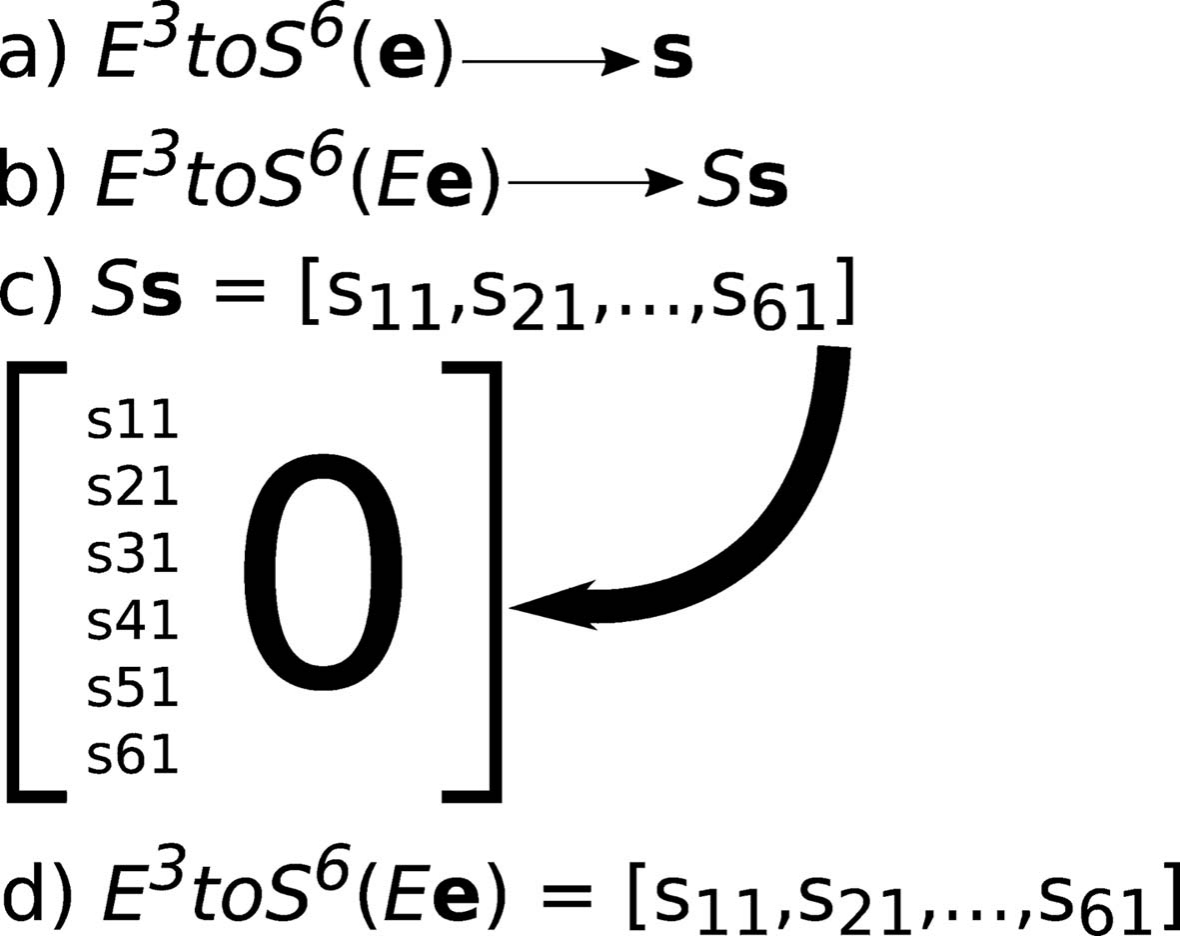
The logic of determining 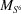. (*a*) *E* ^3^ *toS* ^6^ is an operator that will generate a vector **s** in **S^6^** from a vector **e** in **E** ^**3**×**3**^. (*b*) *E* is a matrix operating on **E** ^**3**×**3**^ and *S* is a matrix operating on **S^6^**. Correspondingly, we can rewrite (*a*) in this more general form. (*c*) Choosing as an example the first basis vector ([1, 0, 0, 0, 0, 0]) in the list of basis vectors, we can then multiply by *S*. The first column of elements of *S* can then be placed into the matrix as indicated. (*d*) In like manner, we can multiply the first basis vector expressed in **E** ^**3**×**3**^ by the matrix *E* in **E^3^** that corresponds to the matrix *S*. However, in this case, the elements of 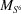 can be computed from the list of calculations above for the first basis vector and the values of matrix *E*. Repeating this process for each of the six basis vectors completes *S*.

As an illustrative example, we choose the first basis vector [above, and matrix **E** in step (*b*) in Fig. 1 ▸],
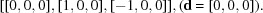
We apply 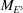 to that vector [step (*a*) in Fig. 1 ▸] and the corresponding 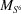 to the corresponding **S^6^** vector. Because only one element of the **S^6^** basis vector is non-zero, the result of multiplying by 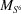 produces only the elements of the corresponding column of 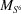, with the other elements being zero [step (*c*) in Fig. 1 ▸]. When we multiply that **E** ^**3**×**3**^ [step (*b*) in Fig. 1 ▸] basis vector by 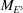 and then convert to **S^6^** using *E* ^3^ *toS* ^6^ [step (*d*) in Fig. 1 ▸], the resulting elements of **S^6^** are the same **S^6^** column elements expressed in terms of the elements of 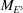.

In each case, only one of the Selling scalars will be −1 and the others will be 0 [step (*c*) in Fig. 1 ▸]. Because **S^6^** is invariant under rotations of **E^3^**, we could have used any unit vector on **E^3^** in place of [1, 0, 0], and we would have obtained the same set of **S^6^** basis vectors.

The computer algebra system *Maxima* (Version 5.36.1; Chou & Schelter, 1986 ▸; http://maxima.sourceforge.net) was used to generate the following equations.

For simplicity, we show the definition of the first row of the **S^6^** matrix (the complete matrix is listed in Section B of the supporting information). For any three-by-three matrix, 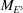, the equation below computes the first column of the negative of the complete matrix (the supporting information has all the columns):
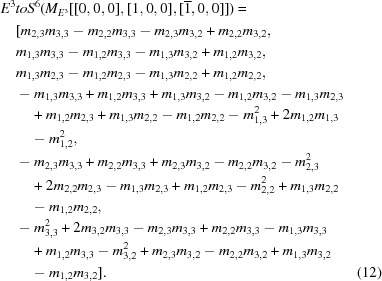



## 4. Conversion of reduced primitive lattices to centered lattices   

Tables 2 ▸ and 3 ▸ list the matrices (Burzlaff & Zimmermann, 1985 ▸) for converting from primitive to standard centered lattices, computed from the above derivations. The designations of the 24 Delone types are slight modifications of the symbols of Delone (1933 ▸) to more modern forms. His cubic lattices are changed from ‘K’ to ‘C’, tetragonal from ‘Q’ to ‘T’ and triclinic from ‘T’ to ‘A’. For each lattice, the **E** ^**3**×**3**^ matrix is listed, followed on the next line by the corresponding **S^6^** matrix.

**Figure 2:**
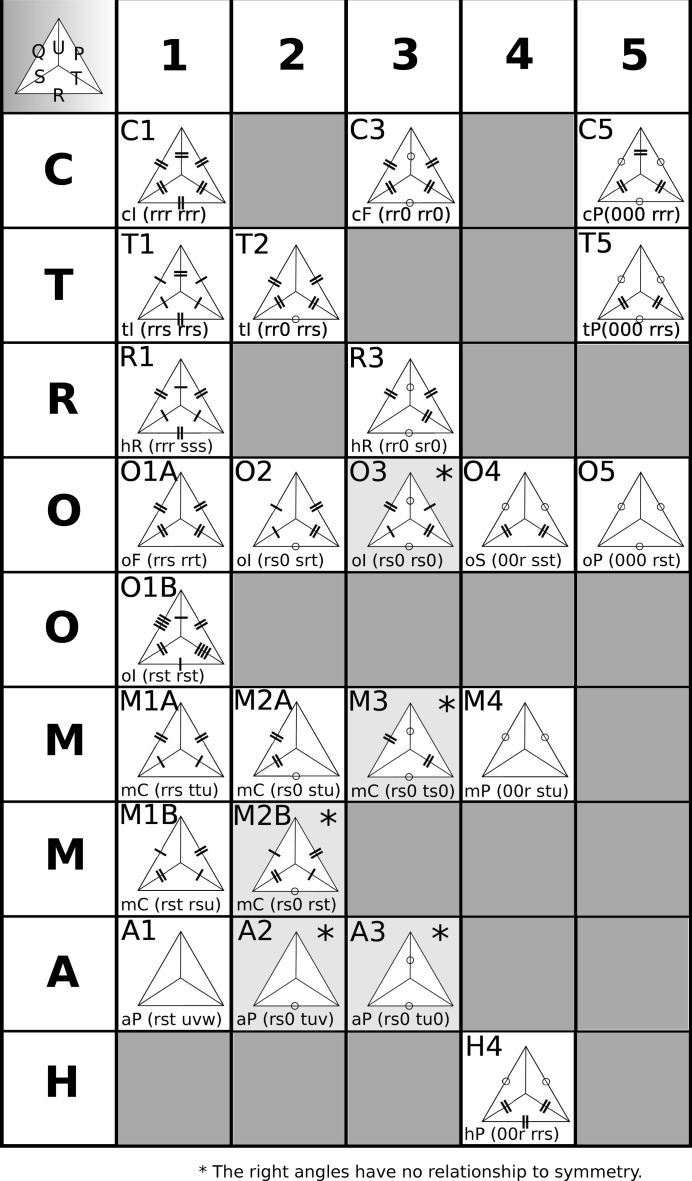
Delone’s table of the 24 canonical types (modified).

Burzlaff & Zimmermann (1985 ▸) renumbered the lattice types of Delone (1933 ▸). For example, the cubic lattices in Delone (1933 ▸) are K1, K3 and K5. In the reports by Burzlaff & Zimmermann (1985 ▸) and Burzlaff *et al.* (1992 ▸), they are listed as K1, K2 and K3. Here they are listed as C1, C3 and C5. The full enumeration of the types is shown in Fig. 2 ▸. It is important to note that Burzlaff *et al.* (1992 ▸) showed the matrices as the transposes of the corresponding matrices of Burzlaff & Zimmermann (1985 ▸). We have chosen to start from the earlier paper. The 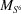 matrices produced are then applied to the left of the **S^6^** vectors. The *International Tables for Crystallography* (Burzlaff *et al.*, 2016 ▸) use the same convention as Burzlaff & Zimmermann (1985 ▸).

## 5. Summary   

This paper is a reference for researchers who need a method that applies to the space **S^6^**, but for which only the matrices applicable to the edge vectors of the unit cell are available. In addition, we have provided a list of the matrices required for conversion of primitive cells in **S^6^** to the more standard centered presentations.

## 6. Availability of code   

The C++ code for distance calculations in **S^6^** is available at github.com, both at https://github.com/duck10/LatticeRepLib.git and https://github.com/yayahjb/ncdist.git For *E* ^3^ *toS* ^6^ see LatticeRepLib/MatS6.cpp.

## Supplementary Material

Discussion of linearity and complete definition of the transformation matrix. DOI: 10.1107/S2053273319014542/ae5074sup1.pdf

## Figures and Tables

**Table 1:** **E** ^**3**×**3**^ basis vectors matched to **S^6^** basis vectors

**E** ^**3**×**3**^ basis vector	**S^6^** basis vector
[[0, 0, 0], [1, 0, 0], 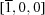 ]	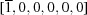
[[1, 0, 0], [0, 0, 0], 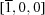 ]	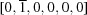
[[1, 0, 0], 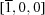 , [0, 0, 0]]	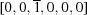
[[1, 0, 0], [0, 0, 0], [0, 0, 0]]	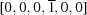
[[0, 0, 0], [1, 0, 0], [0, 0, 0]]	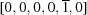
[[0, 0, 0], [0, 0, 0], [1, 0, 0]]	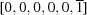

**Table 2:** The first eight of the transformation matrices for each of the 24 Delone types The **E** ^**3**×**3**^ and **S^6^** matrices are both listed in each case. The remaining 16 cases are in Table 3 ▸.

Type	Lattice	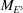 and 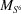
*C*1	*cI*	[[0, 1, 1], [1, 0, 1], [1, 1, 0]]
		[ 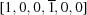 , 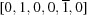 , 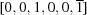 , [0, 0, 0, 0, 2, 2], [0, 0, 0, 2, 0, 2], [0, 0, 0, 2, 2, 0]]
*C*3	*cF*	[[1, 1, 0], 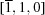 , [1, 1, 2]]
		[ 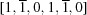 , 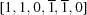 , 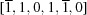 , 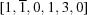 , 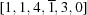 , 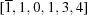 ]
*C*5	*cP*	Identity
*R*1	*hR*	[ 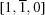 , 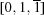 , [1, 1, 1]]
		[ 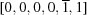 , 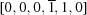 , 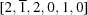 , 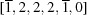 , 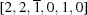 , [0, 0, 0, 2, 1, 0]]
*R*3	*hR*	[[1, 0, 0], [0, 0, 1], [1, 3, 2]]
		[ 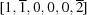 , 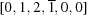 , [0, 1, 0, 0, 0, 0], 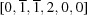 , [0, 1, 0, 0, 0, 3], [0, 1, 2, 2, 9, 6]]
*T*1	*tI*	[[0, 1, 1], [1, 0, 1], [1, 1, 0]]
		[ 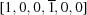 , 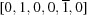 , 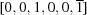 , [0, 0, 0, 0, 2, 2], [0, 0, 0, 2, 0, 2], [0, 0, 0, 2, 2, 0]]
*T*2	*tI*	[[1, 0, 0], [0, 1, 0], [1, 1, 2]]
		[ 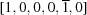 , 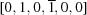 , [0, 0, 1, 0, 0, 0], [0, 0, 0, 2, 0, 0], [0, 0, 0, 0, 2, 0], [0, 0, 0, 2, 2, 4]]
*T*5	*tP*	Identity

**Table 3:** The second 16 of the transformation matrices for each of the 24 Delone types The **E** ^**3**×**3**^ and **S^6^** matrices are both listed in each case. The first eight cases are in Table 2 ▸.

Type	Lattice	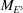 and 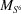
*O*1*A*	*oF*	[[1, 1, 0], 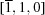 , [1, 1, 2]]
		[ 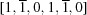 , 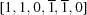 , 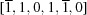 , 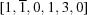 , 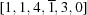 , 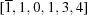 ]
*O*1*B*	*oI*	[[0, 1, 1], [1, 0, 1], [1, 1, 0]]
		[ 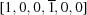 , 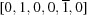 , 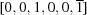 , [0, 0, 0, 0, 2, 2], [0, 0, 0, 2, 0, 2], [0, 0, 0, 2, 2, 0]]
*O*2	*oI*	[[1, 0, 0], [0, 1, 0], [1, 1, 2]]
		[ 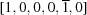 , 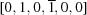 , [0, 0, 1, 0, 0, 0], [0, 0, 0, 2, 0, 0], [0, 0, 0, 0, 2, 0], [0, 0, 0, 2, 2, 4]]
*O*3	*oI*	[[0, 1, 1], [1, 0, 1], [1, 1, 0]]
		[ 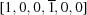 , 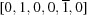 , 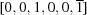 , [0, 0, 0, 0, 2, 2], [0, 0, 0, 2, 0, 2], [0, 0, 0, 2, 2, 0]]
*O*4	*oS*	[ 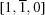 , [1, 1, 0], [0, 0, 1]]
		[[1, 1, 0, 0, 0, 0], 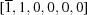 , 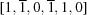 , [1, 1, 4, 2, 0, 0], 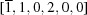 , 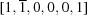 ]
*O*5	*oP*	Identity
*M*1*A*	*mS*	[ 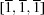 , 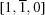 , [0, 0, 1]]
		[ 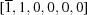 , [0, 0, 0, 0, 0, 1], 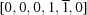 , [0, 0, 0, 0, 2, 0], [2, 0, 4, 0, 2, 0], [2, 0, 0, 0, 0, 0]]
*M*1*B*	*mS*	[[0, 1, 1], [1, 1, 0], 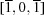 ]
		[ 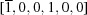 , 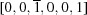 , 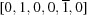 , [0, 0, 2, 0, 2, 0], [2, 0, 0, 0, 2, 0], [2, 0, 2, 0, 0, 0]]
*M*2*A*	*mS*	[ 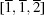 , [0, 1, 0], [1, 0, 0]]
		[[0, 0, 1, 0, 0, 0], 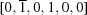 , 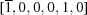 , [2, 2, 0, 0, 0, 4], [2, 0, 0, 0, 0, 0], [0, 2, 0, 0, 0, 0]]
*M*2*B*	*mS*	[[0, 1, 1], [1, 1, 0], 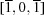 ]
		[ 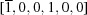 , 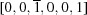 , 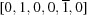 , [0, 0, 2, 0, 2, 0], [2, 0, 0, 0, 2, 0], [2, 0, 2, 0, 0, 0]]
*M*3	*mS*	[ 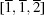 , [0, 1, 0], [1, 0, 0]]
		[[0, 0, 1, 0, 0, 0], 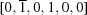 , 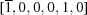 , [2, 2, 0, 0, 0, 4], [2, 0, 0, 0, 0, 0], [0, 2, 0, 0, 0, 0]]
*M*4	*mP*	Identity
*A*1	*aP*	Identity
*A*2	*aP*	Identity
*A*3	*aP*	Identity
*H*4	*hP*	Identity
