# Supplementary material for: Converting three-space matrices to equivalent six-space matrices for Delone scalars in S6
Source: Acta Crystallogr A Found Adv. 2020 Jan 1;76(Pt 1):79–83. doi: 10.1107/S2053273319014542 (PMC7045903; doi:10.1107/S2053273319014542)
Supplement: Supplementary file 1 [file a-76-00079-sup1.pdf]

FOUNDATIONS
ADVANCES

Volume 76 (2020)

Supporting information for article:

Converting three-space matrices to equivalent six-space matrices for Delone scalars in S^6

Lawrence C. Andrews, Herbert J. Bernstein and Nicholas K. Sauter

Converting three-space matrices to equivalent six-space matrices for Delone scalars in S^6 – Supplementary Material

Lawrence C. Andrews,[a]* Herbert J. Bernstein[b] and Nicholas K. Sauter[c]

[a]*Ronin Institute, 9515 NE 137th St, Kirkland, WA, 98034-1820 USA,* [b]*Ronin Institute, c/o NSLS-II, Brookhaven National Laboratory, Upton, NY, 11973 USA, and* [c]*Lawrence Berkeley National Laboratory, 1 Cyclotron Rd., Berkeley, CA, 94720 USA. E-mail: lawrence.andrews@ronininstitute.org*

Lattice Centering; Delaunay; Delone; Niggli; Selling; Matrix transformations

1. Supplementary Material A

In order to understand an important case when T and U are linear even though mappings M_{XY} and M_{YX} are non-linear, consider the simple case where X is the real line, and Y is the non-negative portion of the real line, $M_{XY}(x) = x^2$, and $M_{YX}(y) = \sqrt{y}$. Suppose $T(x) = t\,x$ and suppose $y = x^2 = r\,(x_1^2) + s\,(x_2^2) = r\,y_1 + s\,y_2$. Then

$$M_{XY}(T\,M_{YX}(y_1)) = t^2 x_1^2$$

$$M_{XY}(T\,M_{YX}(y_2)) = t^2 x_2^2$$

$$M_{YX}(y) = \sqrt{y} = \sqrt{r\,y_1 + s\,y_2} = \sqrt{r\,(x_1^2) + s\,(x_2^2)}$$

$$T\,M_{YX}(y) = t\,\sqrt{r\,(x_1^2) + s\,(x_2^2)}$$

$$\Longrightarrow$$

$$M_{XY}(T\,M_{YX}(y)) = t^2\,(r\,(x_1^2)+s\,(x_2^2)) = r\,M_{XY}(T\,M_{YX}(y_1))+s\,M_{XY}(T\,M_{YX}(y_2))$$

which shows that at least in this case U is also linear. Note that our choice of the positive square root for $M_{YX}(y)$ was arbitrary. We could just as well have chosen the negative square root, and the result would have been the same.

2. Supplementary Material B

The computer algebra system, Maxima, version 5.36.1, (Chou & Schelter, 1986) was used to generate the following equations. See [http://maxima.sourceforge.net].

IMPORTANT NOTE: The final line of the function returns the negative of the computed matrix!

```
MatS6 MatS6::e3Tos6(const std::vector<double>& m) {
/*
*************************************************************************
Note that the E3 matrix (m) is applied in the order
shown in Burzlaff and Zimmermann, 1985. This is the
transpose of the order shown in the International
Tables.

The algebra shown here was derived by Herbert Bernstein on 2019-03-19
(with modest help from Larry Andrews). HJB used the program maxima
to do the derivation. Maxima produced the transpose of the matrix
where the S6 vector multiplies from the right, and the values
produced by maxima are the negatives of the matrix coefficients.
*************************************************************************
*/
const double m11 = m[0];
const double m12 = m[1];
const double m13 = m[2];
const double m21 = m[3];
const double m22 = m[4];
const double m23 = m[5];
const double m31 = m[6];
const double m32 = m[7];
const double m33 = m[8];
// %i51) yresult([0, 0, 0], [1, 0, 0], [-1, 0, 0]);

MatS6 m6;
m6[0] = m23 * m33 - m22 * m33 - m23 * m32 + m22 * m32;
m6[6] = m13 * m33 - m12 * m33 - m13 * m32 + m12 * m32;
m6[12] = m13 * m23 - m12 * m23 - m13 * m22 + m12 * m22;
m6[18] = (-m13 * m33) + m12 * m33 + m13 * m32 - m12 * m32
```

```
 - m13 * m23 + m12 * m23 + m13 * m22 - m12 * m22
 - m13 * m13 + 2 * m12*m13 - m12 * m12;
m6[24] = (-m23 * m33) + m22 * m33 + m23 * m32 - m22 * m32
 - m23 * m23 + 2 * m22*m23 - m13 * m23 + m12 * m23
 - m22 * m22 + m13 * m22 - m12 * m22;
m6[30] = (-m33 * m33) + 2 * m32*m33 - m23 * m33
 + m22 * m33 - m13 * m33 + m12 * m33 - m32 * m32
 + m23 * m32 - m22 * m32 + m13 * m32 - m12 * m32;


// (%i52) yresult([1, 0, 0], [0, 0, 0], [-1, 0, 0]);

m6[1] = m23 * m33 - m21 * m33 - m23 * m31 + m21 * m31;
m6[7] = m13 * m33 - m11 * m33 - m13 * m31 + m11 * m31;
m6[13] = m13 * m23 - m11 * m23 - m13 * m21 + m11 * m21;
m6[19] = (-m13 * m33) + m11 * m33 + m13 * m31
 - m11 * m31 - m13 * m23 + m11 * m23 + m13 * m21
 - m11 * m21 - m13 * m13 + 2 * m11*m13 - m11 * m11;
m6[25] = (-m23 * m33) + m21 * m33 + m23 * m31
 - m21 * m31 - m23 * m23 + 2 * m21*m23 - m13 * m23
 + m11 * m23 - m21 * m21 + m13 * m21 - m11 * m21;
m6[31] = (-m33 * m33) + 2 * m31*m33 - m23 * m33
 + m21 * m33 - m13 * m33 + m11 * m33 - m31 * m31
 + m23 * m31 - m21 * m31 + m13 * m31 - m11 * m31;

// (%i53) yresult([1; 0; 0]; [-1; 0; 0]; [0; 0; 0]);

m6[2] = m22 * m32 - m21 * m32 - m22 * m31 + m21 * m31;
m6[8] = m12 * m32 - m11 * m32 - m12 * m31 + m11 * m31;
m6[14] = m12 * m22 - m11 * m22 - m12 * m21 + m11 * m21;
m6[20] = (-m12 * m32) + m11 * m32 + m12 * m31
  - m11 * m31 - m12 * m22 + m11 * m22 + m12 * m21
  - m11 * m21 - m12 * m12 + 2 * m11*m12 - m11 * m11;
m6[26] = (-m22 * m32) + m21 * m32 + m22 * m31
 - m21 * m31 - m22 * m22 + 2 * m21*m22 - m12 * m22
 + m11 * m22 - m21 * m21 + m12 * m21 - m11 * m21;
m6[32] = (-m32 * m32) + 2 * m31*m32 - m22 * m32
 + m21 * m32 - m12 * m32 + m11 * m32 - m31 * m31
 + m22 * m31 - m21 * m31 + m12 * m31 - m11 * m31;

// (%i54) yresult([1, 0, 0], [0, 0, 0], [0, 0, 0]);

m6[3] = m21 * m31;
m6[9] = m11 * m31;
m6[15] = m11 * m21;
m6[21] = (-m11 * m31) - m11 * m21 - m11 * m11;
m6[27] = (-m21 * m31) - m21 * m21 - m11 * m21;
m6[33] = (-m31 * m31) - m21 * m31 - m11 * m31;

// (%i55) yresult([0; 0; 0]; [1; 0; 0]; [0; 0; 0]);

m6[4] = m22 * m32;
m6[10] = m12 * m32;
m6[16] = m12 * m22;
m6[22] = (-m12 * m32) - m12 * m22 - m12 * m12;
m6[28] = (-m22 * m32) - m22 * m22 - m12 * m22;
```

```
m6[34] = (-m32 * m32) - m22 * m32 - m12 * m32;
// (%i56) yresult([0; 0; 0]; [0; 0; 0]; [1; 0; 0]);

m6[5] = m23 * m33;
m6[11] = m13 * m33;
m6[17] = m13 * m23;
m6[23] = (-m13 * m33) - m13 * m23 - m13 * m13;
m6[29] = (-m23 * m33) - m23 * m23 - m13 * m23;
m6[35] = (-m33 * m33) - m23 * m33 - m13 * m33;

for (size_t i = 0; i < 36; ++i) if (m6[i] == 0.0) m6[i] = -0.0;
return -m6;
}
```
